# Data on the clinical, functional, and patient-reported outcomes of patient-centred rehabilitation for patients with non-communicable disease living in low-resourced settings

**DOI:** 10.1016/j.dib.2022.108665

**Published:** 2022-10-08

**Authors:** Martin Heine, Wayne Derman, Ashleigh Müller, Brittany Fell, Mumtaz Abbas, Susan Hanekom

**Affiliations:** a Julius Global Health, Julius Center for Health Sciences and Primary Care, University Medical Center Utrecht, Utrecht, the Netherlands; b Institute of Sport and Exercise Medicine, Faculty of Medicine and Health Sciences, Stellenbosch University, Cape Town, South Africa; c IOC Research Centre, Cape Town, South Africa; d Division of Physiotherapy, Faculty of Medicine and Health Sciences, Stellenbosch University, Cape Town, South Africa; e Bishop Lavis Community Day Centre, Western Cape Department of Health, Cape Town, South Africa

**Keywords:** Developing countries, Randomized clinical trial, Cohort studies, Rehabilitation

## Abstract

In this data article, we present data obtained from a randomized clinical trial aimed at determining the feasibility of patient-centred rehabilitation for people with non-communicable disease (NCD) living in a low-resource setting. Patients were identified at primary care level and considered eligible if having on or more of the NCDs central to the NCD burden of disease (Cardiovascular Disease, Diabetes, Pulmonary Disease or Cancer). Using a “trial within cohort” design, a total 74 patients were included (36% of those identified as eligible) in a longitudinal cohort with repeated assessments at baseline, 8 and 16 weeks. A subset of 50 participants were randomly selected and offered to participate in a 6-week exercise and education-based, minimalistic, community-based rehabilitation program tailored to the low-resource context. The exercise component included aerobic and resistance exercise, as well as thematic empowerment aimed at improving exercise self-efficacy. The education component was aimed at improving general health literacy. Data was collected in terms of feasibility parameters (e.g., uptake, adherence), patient-demographics (e.g., age, gender), medical demographics (e.g., disease burden, multimorbidity), functional capacity measures (e.g., 6-minute Walk Test), and patient-reported outcomes (e.g., health-related quality of life). The data presented can give a basis for further clinical research in this field.

## Specifications Table

**Table:** 

Subject	Physical Therapy and Rehabilitation
Specific subject area	Feasibility of Patient-Centred Rehabilitation
Type of data	Table
How the data were acquired	Data was acquired through physical examination, patient file review, functional exercise tests, and patient-reported outcome measures. Blood pressure, blood glucose, oxygen saturation, heart rate
Data format	Raw Analysed
Description of data collection	Adult patients with one (or more) of four major non-communicable disease clusters, including cancer, cardiovascular disease, pulmonary disease, and diabetes, and without hard contra-indications for exercise therapy.
Data source location	Institute of Sport and Exercise Medicine, Stellenbosch University Cape Town, Western Cape South Africa
Data accessibility	With the article Repository name: Mendeley Data Data identification number: 10.17632/fbhh8dmz7w.1 Direct URL to data: http://dx.doi.org/10.17632/fbhh8dmz7w.1
Related research article	*M. Heine, W. Derman, S. Hanekom,* The "trial within cohort design" was a pragmatic model for low-resourced settings*, J. Clin. Epidemiol.* 147 (2022): 111-121. [1] *M. Heine, W. Derman, S. Hanekom,* Towards a framework for the scale-up of rehabilitation for patients with non-communicable disease in low-resource settings, medRxiv 2022.08.03.22278360. [2] *B. Fell, S. Hanekom, M. Heine.* A modified six-minute walk test (6MWT) for low-resource settings-a cross-sectional study. Heart & Lung. 52 (2022): 117-122. [3]

## Value of the Data

• Data presented in the current paper provide information on a heterogeneous sample of patients with non-communicable disease living in an urban, complex, low-resource setting and can inform future study design and power analyses. These data may be particularly useful for clinical researchers within the rehabilitation space (and beyond) with a focus on resource-constrained settings [4], as they provide real-world insight into within-sample variance and participant flow. Future studies could use these data to make informed decisions with respect to study design, in particular the “Trial within cohort” design, the required sample size, and measures to improve participant adherence and retention.

## 1 Data Description

The data shared are supplementary tables and figures of analysed data from a randomized clinical trial on the feasibility of a minimalistic patient-centred rehabilitation program for people with non-communicable disease living in a low-resource setting [5]. Patients were recruited from a community-based, public-sector, primary care facility within the Cape Flats region of Cape Town, South Africa. The adapted flowchart, published elsewhere [1], outlines the flow of participants through the study, adapted for the “trial within cohort” design adopted [1,^6^,7], and in line with CONSORT recommendations [8]. Data on study uptake, exclusion rate, inclusion rate, intervention uptake, retention at follow-up, and adherence to intervention components can be drawn from this flowchart. Table 1 provides baseline characteristics for the study sample, stratified by the three study arms. Table 2 provides baseline values for the clinical markers, functional measures, and patient-reported outcomes, as well as their values post-intervention and during follow-up. Fifteen data files can be found in the repository that underly each of the tables, including in/exclusion patient characteristics and demographics, medical history, randomisation key, patient-reported outcomes, functional capacity tests, and health economics (patient perspective).

**Table 1:** Patient, medical demographics, and contextual factors for the study sample (N=74) and stratified by study arm.

	Total Sample	Group A (Observational)	Group B (Declined)	Group A + B (Control)	Group C (Intervention)
	*N = 74*	*n = 22*	*n = 18*	*n = 40*	*n = 34*
**Patient demographics**					
*Sex (%Female)*	64	60	72	65	63
*Age, mean (SD)*	59.2 (56.9 to 61.5)	60.0 (55.6 to 64.4)	61.7 (57.4 to 66.9)	60.7 (57.6 to 63.8)	57.4 (54.1 to 60.7)

**Contextual factors**					

*Education, n (%)*					
*- No formal schooling*	3 (4)	2 (9)	0 (0)	2 (5)	1 (3)
*- ≤ Primary school*	24 (32)	3 (14)	6 (33)	9 (23)	15 (43)
*- Grade 8 / 9*	22 (29)	7 (32)	6 (33)	13 (33)	9 (26)
*- Grade 10 / 11*	15 (20)	5 (23)	3 (17)	8 (20)	7 (20)
*- High school completed*	7 (9)	4 (18)	1 (6)	5 (13)	2 (6)
*- College / University*	4 (5)	1 (5)	2 (11)	3 (8)	1 (3)

*Employment, n (%)*					
*- Unemployed (able)*	6 (8)	2 (9)	0 (0)	2 (5)	4 (11)
*- Unemployed (unable)*	21 (28)	5 (23)	4 (22)	9 (23)	12 (34)
*- Employed*	10 (13)	1 (5)	3 (17)	4 (10)	6 (17)
*- Retired*	35 (47)	12 (55)	11 (61)	23 (58)	12 (34)
*- Other*	3 (4)	2 (9)	0 (0)	2 (5)	1 (3)

Annual household income (ZAR)*, n (%)					
*- ≤ 6 485*	3 (4)	0 (0)	2 (11)	2 (5)	1 (3)
*- 6 485 >≤ 13 818*	2 (3)	0 (0)	0 (0)	0 (0)	2 (6)
*- 13 818 >≤ 28 091*	14 (19)	3 (14)	4 (22)	7 (18)	7 (20)
- 28 091 >≤ 71 478	29 (39)	11 (50)	5 (28)	16 (40)	13 (37)
*- > 71 478*	15 (20)	3 (14)	5 (28)	8 (20)	7 (20)
*- Don't know*	12 (16)	5 (18)	2 (11)	7 (18)	5 (14)
*Adults per Household*	3.6 (3.2 to 4.0)	3.9 (3.2 to 4.6)	3.6 (2.7 to 4.5)	3.8 (3.2 to 4.4)	3.4 (2.8 to 4.0)
*Medical insurance, n (%)*	6 (8)	3 (14)	1 (6)	4 (10)	2 (6)
***Risk factors***					
*Smoking, n (%)*	27 (36)	6 (27)	4 (22)	10 (25)	17 (49)

*Alcohol, n (%)*					
*- No consumption <12 months*	48 (64)	13 (60)	11 (61)	24 (60)	24 (69)
*- ≤ once / month*	14 (19)	5 (23)	4 (22)	9 (23)	5 (14)
*- 1 to 3 days / month*	5 (7)	1 (5)	2 (11)	3 (8)	2 (6)
*- 1 to 2 days / week*	6 (8)	2 (9)	1 (6)	3 (8)	3 (9)
*- 3 to 4 days / week*	1 (1)	0 (0)	0 (0)	0 (0)	1 (3)
*- Daily*	1 (1)	1 (5)	0 (0)	1 (3)	0 (0)

*Fruit intake (servings / day)*	0.7 (0.5 to 0.9)	0.8 (0.4 to 1.2)	0.7 (0.3 to 1.1)	0.7 (0.4 to 1.0)	0.6 (0.3 to 0.9)

*Vegetable intake (servings / day)*	0.7 (0.6 to 0.8)	0.7 (0.5 to 1.00)	0.7 (0.4 to 1.0)	0.7 (0.5 to 0.9)	0.6 (0.4 to 0.8)

*Physical activity (IPAQ), n (%)* *- Low* *- Moderate* *- High*	*46 (61)* *20 (27)* *9 (12)*	*14 (64)* *6 (27)* *2 (9)*	*10 (56)* *5 (28)* *3 (16)*	*24 (60)* *11 (28)* *5 (12)*	22 (63) *9 (26)* *4 (11)*
***Medical demographics***					

*Disease profile, n (%)*					
*- Cancer*	2 (3)	1 (5)	0 (0)	1 (3)	1 (3)
*- CVD*	62 (83)	19 (86)	14 (78)	33 (83)	29 (83)
*- Pulmonary disease*	19 (25)	6 (27)	2 (11)	8 (20)	11 (31)
*- Diabetes*	45 (60)	13 (59)	13 (72)	26 (65)	19 (54)
*N comorbidities*	1.7 (1.5 to 1.9)	1.9 (1.5 to 2.3)	1.6 (1.4 to 1.8)	1.8 (1.6 to 2.1)	1.7 (1.5 to 1.9)
***Physiological parameters***					
*Body Mass Index, m^2^/kg*	31.2 (29.5 to 32.9)	31.2 (28.1 to 34.3)	32.1 (28.7 to 35.5)	31.6 (29.3 to 33.9)	30.6 (28.0 to 33.2)
*Waist to hip ratio*	0.93 (0.91 to 0.95)	0.94 (0.90 to 0.98)	0.92 (0.89 and 0.95)	0.93 (0.91 to 0.96)	0.93 (0.90 to 0.96)
*Waist to height ratio*	0.64 (0.62 to 0.66)	0.64 (0.60 to 0.68)	0.65 (0.61 to 0.69)	0.65 (0.62 to 0.68)	0.63 (0.59 to 0.67)
*DBP, mmHg*	80.0 (77.7 to 82.3)	79.8 (75.0 to 84.6)	80.6 (74.8 to 86.4)	80.2 (76.6 to 83.8)	79.7 (76.8 to 82.6)
*SBP, mmHg*	133.5 (130.0 to 137.0)	131.9 (125.0 to 138.0)	139.6 (130.0 to 149.0)	135.3 (130.0 to 141.0)	131.4 (126 to 137)
*RHR, bpm*	78.7 (76.4 to 81.0)	79.9 (76.4 to 83.4)	81.8 (75.7 to 87.9)	80.7 (77.4 to 84.0)	76.3 (73.3 to 79.3)
***Functional measures***					
*6MWT (m)*	380 (363 to 398)	372 (339 to 405)	368 (325 to 411)	370 (344 to 396)	391 (368 to 414)
*TUG (s)*	11.64 (10.60 to 12.60)	11.61 (10.20 to 13.00)	13.38 (9.78 to 17.00)	12.40 (10.60 to 14.20)	10.81 (10.2 to 11.4)
*SSST (left leg; s)*	10.81 (10.00 to 11.60)	10.71 (9.34 to 12.10)	11.58 (9.33 to 13.80)	11.10 (9.85 to 12.30)	10.50 (9.64 to 11.4)
*SSST (right leg; s)*	10.53 (9.66 to 11.40)	10.34 (8.89 to 11.80)	11.23 (8.5 to 14.0)	10.74 (9.28 to 12.20)	10.31 (9.43 to 11.2)
***PROMs^#^***					
*PSQI (global score; range 0 - 21)^‡^*	7.7 (2.4 to 5.9)	8.3 (6.9 to 9.7)	7.2 (5.3 to 9.1)	7.8 (6.6 to 9.0)	7.5 (6.0 to 9.0)
*EQ-5d (general health; range 0 - 100)*	73.0 (67.9 to 78.1)	68.2 (58.0 to 78.4)	73.1 (62.8 to 83.4)	70.4 (63.2 to 77.6)	76.0 (68.8 to 83.2)
*EQ-5d (health state; range 0 - 1.00)*	0.86 (0.81 to 0.91)	0.85 (0.73 to 0.97)	0.82 (0.71 to 0.93)	0.84 (0.76 to 0.92)	0.88 (0.83 to 0.93)
*IPAQ (METs / week)*	1099 (723 to 1480)	1035 (405 to 1670)	1238 (272 to 2200)	1126 (577 to 1680)	1067 (555 to 1580)

Values are provided as n (%) for count variables and mean (95% confidence intervals) for continuous variables unless indicated otherwise. 6MWT, 6-Minute Walk Test; CVD, Cardiovascular Disease; DBP, Diastolic Blood Pressure; EQ-5d, EuroQol-5 Dimension general health score and health state (based on Ethiopian reference set); IPAQ, International Physical Activity Questionnaire; METs, Metabolic Equivalents; PSQI, Pittsburgh Sleep Quality Index; PROMs, Patient-reported Outcome Measures; RHR, Resting Heart Rate; SBP, Systolic Blood Pressure; SSST, Six-Spot Step Test; TUG, Timed Up & Go test; ZAR, South African Rand. *Average provincial household income (Census 2011) is 143 460 ZAR/year. ^#^Due to the limited amount available, data for the productivity cost questionnaire will be presented elsewhere^‡^ Lower score is a better outcome. Modified from Heine et al. [1].

**Table 2:** Data for key clinical, functional, and patient-reported outcomes for various assessment points.

	Baseline		8wk Follow-Up		16wk Follow-Up	
	Control	Intervention	Control	Intervention	Control	Intervention
***Clinical markers***	***40***	***34***	***21***	***19***	***22***	***13***
*Body Mass Index, m^2^/kg*	31.6 (29.3 to 33.9)	30.6 (28.0 to 33.2)	*31.3 (28.2 to 34.4)*	*30.2 (26.5 to 33.9)*	*31.7 (28.8 to 34.6)*	*30.6 (26.8 to 34.4)*
*Waist to hip ratio*	0.93 (0.91 to 0.96)	0.93 (0.90 to 0.96)	*0.94 (0.91 to 0.97)*	*0.94 (0.91 to 0.97)*	*0.92 (0.89 to 0.95)*	*0.94 (0.90 to 0.98)*
*Waist to height ratio*	0.65 (0.62 to 0.68)	0.63 (0.59 to 0.67)	*0.65 (0.61 to 0.69)*	*0.63 (0.58 to 0.68)*	*0.64 (0.61 to 0.67)*	*0.64 (0.59 to 0.69)*
*SBP, mmHg*	80.2 (76.6 to 83.8)	79.7 (76.8 to 82.6)	*138 (129 to 147)*	*132 (127 to 137)*	*136 (129 to 143)*	*141 (133 to 149)*
*DBP, mmHg*	135.3 (130.0 to 141.0)	131.4 (126.0 to 137.0)	*85 (81 to 89)*	*80 (76 to 84)*	*82 (78 to 86)*	*81 (76 to 86)*
*RHR, bpm*	80.7 (77.4 to 84.0)	76.3 (73.3 to 79.3)	*82 (76 to 88)*	*78 (73 to 83)*	*82 (77 to 87)*	*77 (69 to 85)*
						
***Functional measures, n valid***	***40***	***34***	***17***	***19***	***17***	***12***
*6MWT (m)*	370 (344 to 396)	391 (368 to 414)	*361 (331 to 391)*	*381 (353 to 409)*	*386 (356 to 416)*	*391 (370 to 413)*
*TUG (s)*	12.40 (0.6 to 14.2)	10.81 (10.20 to 11.40)	*10.90 (10.3 to 11.5)*	*11.96 (10.80 to 13.10)*	*11.19 (10.6 to 11.7)*	*11.54 (10.40 to 12.60)*
*SSST (s) – Left leg*	11.10 (9.85 to 12.3)	10.50 (9.64 to 11.40)	*11.17 (8.7 to 13.7)*	*10.53 (9.47 to 11.60)*	*9.93 (9.04 to 10.8)*	*10.21 (9.34 to 11.10)*
*SSST (s) – Right leg*	10.74 (9.28 to 12.20)	10.31 (9.4 to 11.2)	*11.14 (9.1 to 13.3)*	*10.24 (9.31 to 11.20)*	*9.76 (8.9 to 10.7)*	*9.80 (9.03 to 10.6)*
***PROMs^#^***	***40***	***34***	***22***	***21***	***22***	***13***
*PSQI (global score; range 0 - 21)*	7.8 (6.6 to 9.0)	7.5 (6.0 to 9.0)	*6.6 (5.2 to 8.0)*	*6.1 (4.0 to 8.2)*	*6.8 (5.5 to 8.1)*	*6.9 (4.9 to 8.9)*
*EQ-5d (general health; range 0 - 100)*	70 (63.2 to 77.6)	76 (68.8 to 83.2)	*80 (72.3 to 87.7)*	*79 (73 to 85)*	*77 (70 to 85)*	*72 (56 to 88)*
*EQ-5d (health state; range 0 – 1)*	0.84 (0.76 to 0.92)	0.88 (0.83 to 0.93)	*0.854 (0.768 to 0.940)*	*0.899 (0.833 to 0.965)*	*0.873 (0.795 to 0.951)*	*0.875 (0.818 to 0.932)*
*IPAQ (METs / week)*	1126 (577 to 1680)	1067 (555 to 1580)	*836 (352 to 1320)*	*1183 (724 to 1640)*	*1938 (808 to 3070)*	*1485 (395 to 2580)*

Values are provided as mean and 95% confidence intervals; 6MWT, 6-minute walk test; EQ-5d, EuroQol- 5 Dimension general health score and health state (based on Ethiopian reference set); DBP, Diastolic Blood Pressure; IPAQ, International Physical Activity Questionnaire; METs, Metabolic Equivalents; PSQI, Pittsburgh Sleep Quality Index; RHR, Resting Heart Rate; SBP, Systolic Blood Pressure; SSST, Six Spot Step Test; TUG, Timed Up and Go test.

## 2 Experimental Design, Materials and Methods

A detailed description of the intervention and outcomes is provided elsewhere [5]. Herein, a short description is provided.

### 2.1 Population

Adult patients with non-communicable disease (i.e., Cancer, Cardiovascular Disease, Chronic Pulmonary Disease and/or Diabetes) were included from a public-sector primary care facility. Comorbidities were considered unless they were considered a strict safety-risk for participating in exercise therapy, as per the guidelines provided by the American College of Sports Medicine [9], and outline in the study protocol [5]. Furthermore, patients were excluded if they would jeopardize the safe conduct of study, for example severe psychiatric disease or irrational behaviour related to drug or alcohol abuse.

### 2.2 Intervention

Participants in the intervention group were offered a 6-week patient-centred rehabilitation program comprising of:

i) One supervised session a week of individualised aerobic and resistance training presented in a group setting. Each of the 6 exercise sessions had a specific theme (e.g., safety of exercise, what comprises moderate intensity, goal setting) to promote exercise self-efficacy. Individual exercise prescription was based on baseline assessment (e.g., outcome of the 6-minute walk test), and completed by a clinical physiotherapist, experienced in providing group-based interventions in low-resource settings. No specialized equipment was used, rather easily accessible resources (e.g., water bottle),to ensure continuation of the program at home, twice per week. The overarching aim was to equip the participants to be active for 150 min/week at a moderate intensity following completion of the program.
ii) Every other week, participants would engage in a one-hour health education session covering the themes of non-communicable diseases related to lifestyle, heart-health behaviour, and the benefits of physical activity. Sessions were facilitated by the physiotherapist.

As programs as short as 3 – 5 weeks have shown clinical benefits [5], in this study, a six-week program was hypothesised to impact clinical, functional and/or patient-reported outcomes, while minimizing the resources required (provider, and patient).

Session adherence was recorded by the supervising physiotherapist (AR), and home-based exercise was recorded by participants in an exercise diary. All patients received care as usual, including routine follow-up health screening by the primary physician. The control group did not receive structured exercise and education.

### 2.3 Outcome measures

A detailed description of the outcome measures included in the study are provided elsewhere [5]. In the present report, the following sample characteristics and outcome measures are included.

• Patient demographics (sex, age)
• Contextual factors (education level, employment, household income, number of adults per household, and number of participants with medical insurance)
• Medical demographics (Number of people with cancer, cardiovascular disease, pulmonary disease and/or diabetes, and number of comorbidities)
• Risk factors (smoking, alcohol, fruit and vegetable intake, physical activity)
• Clinical markers (body mass index, waist-to-hip ratio, waist-to-height ratio, systolic blood pressure, diastolic blood pressure, and resting heart rate)
• Functional measures (6-minute walk test, timed up-and-go test, six-spot step test)
• Patient-report outcome measures (Pittsburgh Sleep Quality Index, EuroQol 5D measure of Health-Related Quality of Life, and the International Physical Activity Questionnaire)

Repeated measures are presented for the clinical, functional, and patient-reported outcomes.

### 2.4 Trial within cohort design, randomization, and masking

A concealed, decentralized, variable block-size randomization (3:1) scheme was used as part of a trial within cohort design meaning that all participants were recruited for a longitudinal cohort with assessments at baseline, 8 weeks, and 16 weeks. Subsequently, 75% of participants were offered to participate in the experimental intervention. Participants could subsequently make an informed decision whether they wanted to participate or not. Reasons for non-participation were noted, and since those participants remained part of the cohort, detailed health outcomes were obtained. Those participants that were not offered the intervention remained in the cohort and received care as usual. All outcomes were assessed blinded to treatment allocation by a trained physiotherapist (BF).

### 2.5 Statistical analyses

Due to the small sample size and lack of a clearly operationalised null hypothesis, no formal statistical analyses were conducted, or p-values provided. Data are presented as mean and 95% confidence intervals. Future studies need to make an informed decision with respect to the type of analysis most appropriate for their respective study, including per-protocol analyses, complier average causal effect analyses, or conventional intention-to-treat analyses. Similarly, an informed decision can be made with respect to participants unwilling to consent to the experimental condition whether to treat those as controls, or as part of the treatment group.

## Ethics Statements

This trial was prospectively registered (PACTR201807847711940). Ethical approval was obtained from the Stellenbosch University Health Research and Ethics council (HREC2-2018-0913), and Western Cape Health Research Committee (WC-201806-027). All participants provided oral and written informed consent.

## CRediT Author Statement

**Martin Heine:** Conceptualization, Methodology, Formal Analyses, Writing – original draft, Visualization, Supervision, Project administration, Funding acquisition; **Wayne Derman:** Conceptualization, Writing – review & editing; **Ashleigh Müller:** Investigation, Writing – review & editing; **Brittany Fell:** Writing – review & editing, Investigation; **Mumtaz Abbas:** Writing – review & editing, Investigation; **Susan Hanekom:** Conceptualization, Writing – review & editing.

## Declaration of Competing Interest

The authors declare that they have no known competing financial interests or personal relationships that could have appeared to influence the work reported in this paper.

The authors declare the following financial interests/personal relationships which may be considered as potential competing interests.

## Data Availability

Dataset for the Feasibility of Patient-Centred Rehabilitation in Low-resource Settings (Original data) (Mendeley Data).
